# Alertness and visuospatial attention in clinical depression

**DOI:** 10.1186/1471-244X-11-78

**Published:** 2011-05-09

**Authors:** Lisa Schock, Michael Schwenzer, Walter Sturm, Klaus Mathiak

**Affiliations:** 1 Department of Psychiatry, Psychotherapy and Psychosomatics, Medical School, RWTH Aachen University, Aachen, Germany; 2 JARA - Translational Brain Medicine, Jülich, Germany; 3 Department of Neurology, Clinical Neuropsychology, Medical School, RWTH Aachen University, Aachen, Germany; 4 Institute for Neuroscience and Medicine, INM-1, Research Centre Jülich, Jülich, Germany

## Abstract

**Background:**

Cognitive deficits are a substantial burden in clinical depression. The present study considered dysfunction in the right-hemispheric attention network in depression, examining alertness and visuospatial attention.

**Methods:**

Three computerized visuospatial attention tests and an alertness test were administered to 16 depressive patients and 16 matched healthy controls.

**Results:**

Although no significant group effect was observed, alertness predicted reduced visuospatial performance in the left hemifield. Furthermore, sad mood showed a trend towards predicting left visual field omissions.

**Conclusions:**

Decreased alertness may lead to lower left hemifield visuospatial attention; this mechanism may be responsible for a spatial bias to the right side in depression, even though treatment of depression and anxiety may reduce this cognitive deficit.

## Background

It is well documented that depressive disorders are associated with cognitive deficits, including problems with attention, executive functions, and memory [1-4]. Cognitive symptoms are often considered by-products of depression, and expected to diminish with clinical improvement. For many patients, however, cognitive impairments represent a primary concern and are noted to chronically impair activities of daily living [5]. For example, visuospatial neglect can interfere with navigation in space - one of our clinical cases reported falling into the installation pit of his workshop when passing on the right-hand side. In this individual, in addition to hemineglect, moderate to severe depressive scores were reported, and then, with remission of the depression, neuropsychological deficits also subsided. In the present study, we focused on visuospatial deficits in depression and its relation to alertness and mood symptoms.

With regard to visuospatial attention, behavioural studies have reported left visual field impairments in depressive patients [6,7], as well as in healthy subjects during conditions of induced sadness [8-10]. Different interpretations have been offered to explain these lateralization effects. According to Banich and colleagues, depression might be associated with hyperactivity in right frontal regions and hypoactivity in posterior regions, resulting in attention problems [10]. Higher activation in the right hemisphere, particularly in frontal regions, was found to be indicator of negative affect in healthy populations [11]. Liotti and Mayberg suggested a model in which limbic activation in sadness leads to deactivation of cortical sites involved in the processing of attention stimuli, such as dorsolateral prefrontal cortex and inferior parietal cortex. In fact, depression was found to interact with right-hemisphere dorsal cortico-limbic networks mediating space processing and externally directed attention and arousal [3].

Classically, visuospatial neglect has been observed after brain lesions, predominantly in the left visual field after right-hemisphere lesions. Patients "ignore" stimuli in the visual field contralateral to the lesion and, as such, they fail to properly interact with and navigate their environment. In laboratory measures, visuospatial neglect is reflected by prolonged reaction time to and omissions of laterally presented stimuli in the affected visual field [12,13]. Current neuropsychological models of visuospatial neglect are based on a strong association of alertness with visuospatial impairments [14]. Evidence was also provided by Thimm and colleagues who showed that alertness training in patients with right-hemisphere lesions and left-sided visuospatial neglect resulted in a transient amelioration of neglect symptoms on the behavioural level as well as to reactivation of associated brain areas. Thus, alertness seems to be a necessary but not sufficient factor of normal functioning of visuospatial attention [15]. Sturm and colleagues investigated the functional anatomy in an intrinsic alertness and a visuospatial attention task and found a combined right-hemispheric network in the prefrontal and inferior parietal cortex [16]. This functional connection may account for the finding that lesions in these areas elicit an alertness deficit associated with visuospatial deficits. However, it is unknown if this mechanism applies in depression as well.

The aim of the present study was to investigate the relation of visuospatial attention in depression to sad mood and alertness deficits, examining performance on standard computer measures for visuospatial attention and alertness in a group of patients diagnosed with depression and in a group of matched controls (see methods section). Sad mood was assessed using the Beck Depression Inventory (BDI) [17]. Based on the literature, our first goal was to confirm left hemifield visuospatial impairments in individuals with depression (reflected as a rightward spatial bias). Our second goal was to measure whether alertness deficits or sad mood would enhance such an attention bias.

## Methods

### Subjects

Sixteen patients and 16 controls pair-wise matched on gender, age, and education were examined. All patients were diagnosed with major depressive episode according to DSM-IV-TR [18] and ICD-10 (F 32, F 33) [19]. According to the Edinburgh Handedness Inventory [20], 14 out of the 16 patients were right-handed and 2 were ambidextrous; 15 of the healthy controls were right-handed and one was ambidextrous. Psychopathology was measured using Hamilton Depression Scale [21] in depressive patients as well as Beck Depression Inventory [17] and State-Trait Anxiety Inventory [22] in both groups. Table 1 lists the demographic characteristics for each group. Patients were recruited from the in- and outpatient services of the Department of Psychiatry, Psychotherapy and Psychosomatics at the RWTH Aachen University. All patients were treated with antidepressant medication, mainly selective serotonin or noradrenalin reuptake inhibitors and tricyclic antidepressants. Additionally, four patients received atypical antipsychotics, and four were medicated with a benzodiazepine. The study was approved by the local Ethics Committee of the RWTH Aachen University. Informed consent was given by all patients and healthy controls prior to participation in the study.

**Table 1:** Demographic characteristics of the sample (mean ± SD)

	Depression (n = 16)	Control (n = 16)	T (df = 15)	p
Age	44.7 ± 9.1	45.3 ± 9.6	-1.04	.314
Gender (f:m)	10:6	10:6	-	-
Years of education	16.8 ± 5.1	17.8 ± 4.1	-1.19	.252
HAMD	25.4 ± 9.3	-	-	-
BDI				
Total score	17.1 ± 9.5	3.3 ± 4.4	6.04	.000
STAI X1	45.9 ± 9.8	35.1 ± 8.2	3.77	.002
STAI X2	53.8 ± 12.4	32.5 ± 8.9	5.62	.000
Number of depressive episodes	1.9 ± 0.9	-	-	-
Days since treatment onset for current episode	69.1 ± 32.5	-	-	-

HAMD: Hamilton Depression Scale, BDI: Beck Depression Inventory, STAI: State-Trait Anxiety Inventory (X1: state, X2: trait); number of depressive episodes were counted as number of in-hospital treatments.

### Neuropsychological assessment

Visuospatial deficits were assessed by the number of omissions of stimuli presented to each visual field. Three different computer visuospatial neglect tests were administered: the Neglect and Visual Scanning subtests of the Test Battery for Attention Performance TAP 2.1 [23] and the Extinction-Neglect (Spatial Attention) subtest of the Test Battery for Perception and Attention Functions (WAF) [24] of the Vienna Test System. The computerized alertness test was the WAF subtest Alertness intrinsic visual [25] (a simple visual reaction time task without warning stimuli), also from the Vienna Test System [26]. Subjects were seated in a sound-proof room at a distance of 50 cm from a 38 × 30 cm computer screen.

#### TAP, subtest Neglect

Subjects fixated on a central square (size about 1.6°) on a black screen. For constant fixation subjects had to read out loud single letters that appeared inside the square and that changed every few seconds. Surrounding the square, small two- and three-digit numbers serving as distractors were randomly distributed over the screen, 24 in each visual field. The distractors may have had an enhancing effect on potential neglect symptoms by provoking "extinction" phenomena in the neglected hemifield. In between these distractor stimuli target stimuli appeared within about 19.5° of the central square in the left or right visual field. Targets were flickering three digit numbers. Subjects were instructed to press the key with the right hand as fast as possible whenever a target appeared. The target stimulus disappeared with the key press or if no response occurred after 3 sec. The 22 targets were presented in each visual field at random locations. The dependent variable was the number of omissions in each visual field. The odd-even-reliability of the test is known to be r = 0.92-0.95; normative data of a representative population of n = 200 persons from age 20 to 69 are available. Validity of the test is confirmed by the diagnosis of visuospatial impairments in neurological patients [27,28]. Median reaction times were analyzed to control for speed-accuracy trade-off.

#### TAP, subtest Visual Scanning

Subjects were to detect a target stimulus consisting of a square with a gap at its top in a 5 × 5 matrix of similar distractors with gaps to the right, to the left, or at the bottom. 100 matrices were presented, half of them containing a target stimulus. Target stimuli randomly appeared twice at each position. Subjects were instructed to scan the matrix as fast as possible from the top left to the bottom right and to decide whether or not the matrix contained a target stimulus by pressing either a left ("yes") or right ("no") response key. The dependent variable was the number of omissions in the leftmost and rightmost column. The odd-even-reliability of the test is known to be r = 0.89; normative data of a representative population of n = 397 persons from age 19 to 90 are available. Validity of the test is confirmed by the diagnosis of visuospatial neglect in neurological patients [27,28]. Median reaction times were analyzed to control for speed-accuracy trade-off.

#### WAF, subtest Extinction-Neglect

Stimuli were presented at different positions in the left or right visual field, or simultaneously at equivalent positions in both hemifields in the extinction condition. Stimulus duration was 3 seconds with an inter-stimulus interval of 3-5 seconds. Subjects were instructed to press the button "5" on the keyboard with the index finger of the right hand as soon as a small dot appeared in the left hemifield, and the button "6" with the middle finger of the right hand for a target dot in the right visual field. They were instructed to fixate on a dot with a cross that was presented in the middle of the screen throughout the task. In the extinction condition, both buttons had to be pressed simultaneously. Number of omissions summated across the uni- and bilateral condition for each visual field served as dependent variables. The reliability of the test was found to be r = 0.93 (Cronbach's alpha); normative data of a representative population of n = 283 persons from age 16 to 88 are available. The test assesses spatial attention distribution based on the model of Posner [29]. Mean reaction times were analyzed to control for speed-accuracy trade-off.

#### WAF, subtest Alertness intrinsic visual

Subjects were instructed to fixate on a cross in the center of the screen and to press a button as fast as possible with their right hand as soon as a black dot appeared in the center of the screen. The stimulus duration was 1.5 seconds and the inter-stimulus interval was 3-5 seconds. The dependent variable was the mean reaction time. The reliability of the test is known to be r = 0.93 (Cronbach's alpha); normative data of a representative population of n = 295 persons from age 16 to 77 are available. Measures for intrinsic alertness reflect the intensity aspect of attention, the response readiness without any external preparatory cue [30].

### Statistical analysis

Visuospatial impairments concerning the left hemifield are represented by a relative preponderance of omissions and higher reaction time values in the left visual field compared to the right visual field, which results in positive values when calculating left minus right visual field performance. A difference score was computed for each subtest. Alertness performance was measured by reaction time. General attention deficits, partly assessed by the WAF Alertness subtest, would be observed as prolonged reaction times in the patients. As such, we instead focused on the difference measure of omissions in the left versus right hemifield to reduce confounds. To assess the first hypothesis, paired t-tests compared the lateralization scores of patients with those of controls. The second hypothesis was tested in a linear regression model with the predictors BDI sad mood item and WAF Alertness across both groups within a repeated-measures design. Moreover, a multivariate ANOVA explored the influence of gender on performance in all four tests. Significance level was .05 two-sided, corrected for the three tests according to Bonferroni.

## Results

Comparisons of the neglect tests concerning omissions as well as reaction time and of the alertness test yielded no significant differences between depressive patients and healthy controls.

The difference scores of omissions in the left visual field and those in the right visual field, however, yielded higher values in the depression group in all three tests, indicating a bias in the direction of the right visual field. Table 2 shows the comparisons in detail. Concerning omissions in TAP Neglect, depressive patients were biased to the right visual field and healthy controls to the left visual field. In the TAP Visual Scanning task, depressive patients and healthy control subjects both showed negative values for omissions, indicating a bias to the left hemispace. In the WAF Extinction-Neglect subtest, depressive patients had a rightward bias (higher omission rate in the left visual field) whereas omissions tended to a value of zero in controls. Reaction time in WAF alertness showed no significant difference between depressive patients and healthy controls, although an increased variance in the depression group is notable.

**Table 2:** Visual field difference of number of omissions and Alertness performance (mean ± SD)

	Depression (n = 16)	Control (n = 16)	T (df = 15)	p	regression: **total R**^**2**^	p	β1: WAF alertness β2: BDI sad mood	T (df = 13)	p
TAP Neglect									
Omissions (LVF-RVF)	0.19 ± 0.75	-0.21 ± 0.89	1.24	.234	.458	.019	β1: .67 β2: -.12	3.27 -0.59	.006* .564
TAP Visual Scanning									
Omissions (LVF-RVF)	-0.25 ± 2.27	-0.71 ± 2.59	0.48	.638	.047	.731	β1: -.19 β2: .10	-0.71 0.38	.491 .707
WAF Extinction-Neglect									
Omissions (LVF-RVF)	0.50 ± 1.10	0.06 ± 0.93	1.39	.186	.369	.050	β1: .40 β2: .45	1.80 2.06	.095 .060
WAF Alertness									
(RT in ms)	278.8 ± 100.3	261.0 ± 29.2	0.68	.510	-	-	-	-	-

LVF: left visual field, RVF: right visual field, RT: reaction time, *significant according to Bonferroni-corrected threshold; T-values and p-values of the group comparisons are displayed together with parameters of a linear regression model including the predictors WAF Alertness and sad mood item of BDI.

The results of the linear regression analysis showed that the combination of alertness performance and subjective rating of sad mood predicted performance on visuospatial attention tests. The factor WAF Alertness had a significant influence on the omissions in TAP Neglect, with longer reaction times predicting a rightward bias (β1 = .67, T_13 _= 3.27, p < .006). The relation of alertness and rightward bias in WAF Extinction-Neglect showed a trend in the same direction (β1 = .40, T_13 _= 1.80, p < .095). In the TAP Visual Scanning subtest, neither alertness nor sad mood influenced the number of omissions. Omissions in TAP Neglect were not influenced by sad mood. Sad mood in the BDI showed a trend towards predicting a rightward bias in WAF Extinction-Neglect (β2 = .45, T_13 _= 2.06, p < .060). For detailed results see Table 2.

Arguing against a speed-accuracy trade-off, the mean reaction time differences of left and right visual field (± SD, in ms) revealed similar tendencies in both groups in contrast to omission differences. Slightly longer reaction times emerged in the left visual field during the TAP Neglect test (depression: 8.19 ± 53.19, control: 24.86 ± 95.14) and shorter reaction times in the left visual field during both TAP Visual Scanning (depression: -373.88 ± 1274.87, control: -350.64 ± 1169.09) and WAF Extinction-Neglect (depression: -33.44 ± 50.53, control: -31.71 ± 29.10). Reflecting the increased variability of the natural sample, high variances were noted in both groups. Testing for further covariates, no significant effect emerged concerning the influence of gender on performance in alertness and visuospatial attention tests (F [7,6] = 0.568, p < .762).

## Discussion

The first hypothesis predicted visuospatial impairments in the left visual field in individuals with depression, reflected as a bias to the right visual field. A statistically significant difference of lateralized attention in patients versus controls, however, was not established. Our second hypothesis predicted a relationship between sad mood, alertness and a left visuospatial attention deficit in depressive patients. Our data supported the view that alertness deficits in depression promote visuospatial impairment in the left hemifield, reflected as a rightward bias of spatial attention, whereas sad mood showed a trend in the same direction. The influence of alertness on visuospatial attention was hypothesized, based on the model of a combined network of alertness and visuospatial attention [15,16]. Moreover, the effect of mood - although weak - also supports the hypothesis that sadness inhibits visuospatial attention processing in the right hemisphere and therefore favours left visual field impairment [3].

Our patient sample yielded a trend towards left-sided visuospatial deficits, seen as positive values in the number of omissions in two of the three neglect tests (i.e., an attention bias towards the right visual field). Since reaction times revealed no group difference concerning the visual field bias in all three tests, speed-accuracy trade-off could not account for different tendencies in omission scores. Moreover, reaction times must be considered as possible confounds of alertness differences in both groups and should therefore not be interpreted with regard to our hypotheses.

There are, indeed, several reasons that may explain the absence of stronger (and significant) cognitive deficits in the group as a whole. In the present study, mean treatment duration of the patients was longer than two months at testing time. Cognitive disturbances thus may have been reduced by antidepressant medication. When testing acutely depressed patients, a different cognitive profile may be expected. Grant and co-workers administered a neuropsychological test battery to a group of unmedicated middle-aged mildly depressed patients and showed that reaction time was positively correlated to illness severity and that there was a moderate relationship of illness severity and performance deficit in attention shifting [31]. As our sample comprised mildly to moderate depressed patients, low illness severity may explain the lack of significant differences between depressive patients and healthy controls.

Concerning arousal, whereas depression is commonly associated with low arousal, anxiety is associated with high arousal. On the neural level, the right posterior region is hypothesized to exhibit lower activation in depression and higher activation in anxiety [11]. According to the spatial attention network model, these different levels of arousal may influence anterior regions in the same way [16]. Comorbid anxiety in depression may therefore abolish the low arousal and lead to increased activity in the attention network. The fact that depressive patients scored significantly higher in state and trait anxiety than our healthy subjects may have contributed to the relative high values of arousal and consequently may have prevented observing a more pronounced visuospatial attention deficit in the current sample.

Omission scores on the TAP Neglect subtest were predicted by alertness. In the WAF Extinction-Neglect subtest, alertness and sad mood influenced omission scores at a trend-level. Using three different tests and a conservative correction for multiple testing reduced the statistical power in the current study. Furthermore, we noted a degree of variability across the tests.

It has to be taken into account that different visuospatial attention measures differ in sensitivity and recruitment of processing resources. Reviewing several studies on cognitive impairment in depression, Levin and colleagues concluded that the problems arise from using a different strategy in the allocation of resources [32]. In the present study, this finding may be reflected by a more rightward attention bias in the TAP Neglect and WAF Extinction-Neglect subtests in depressive patients. These two tests comprised a restriction for the time to answer a stimulus, whereas in TAP Visual Scanning the participant was instructed to answer as fast as possible but nevertheless stimulus set changed with the participants' button press. More time could be taken to scan the stimulus set and thus less processing resources may have been needed.

The leftward bias in control subjects observed in two of the three neglect tests may reflect a phenomenon termed pseudoneglect, originally investigated in line bisection tasks and inducing a leftward bias in neurologically healthy subjects. This effect seems to be substantially dependent on the scanning strategy (left-to-right or right-to-left) [33], and could thereby be another explanation for the leftward bias of depressive patients in the visual scanning task, where a left-to-right scanning strategy is part of the task. The cognitive control of attention distribution is also an important factor in the rehabilitation of visuospatial neglect in stroke patients [34,35]. Accordingly, the use of a left-to-right scanning strategy in the subtest TAP Visual Scanning may explain the absence of a comparable trend to a rightward bias seen in the other two tests in depressive patients.

The rather small effect of sad mood on the attention bias may be explained by the restricted validity of the sad mood item of the BDI. When considering the complete BDI score - a well established measure of depressiveness - a stronger effect concerning the relation of attention and depressiveness may have occurred. In our hypothesis, however, we emphasized the influence of sad mood on alertness and spatial attention deficits. Depressiveness includes a wide range of symptoms, including feelings of guilt, reduced drive and suicidal thoughts, thus not only negative emotion as relevant for our hypothesis. A questionnaire measuring negative emotions such as the Positive and Negative Affect Schedule (PANAS) [36] may be a more useful tool in future studies to investigate the influence of negative emotion on cognitive functioning in depressive disorder.

## Conclusions

In our sample, depressive patients did not show significant spatial attention deficits in the left visual field as compared to controls. However, we confirmed the hypothesis that alertness deficits and at a trend level subjectively experienced sadness in depression enhanced a rightward bias of spatial attention. We suggest that reduced alertness is a mechanism that can lead to visuospatial impairments in depression. Stimulus-driven spatial attention tasks seem to be more suitable for the analysis of visuospatial impairments in depressive patients as compared to tasks involving strategy use. Since cognitive deficits, particularly attention impairments, can substantially reduce quality of life, future studies should also investigate impaired attention and the underlying mechanisms in psychiatric disorders. Importantly, the interaction between attention mechanisms and disturbed emotions requires further analysis.

## Competing interests

The authors declare that they have no competing interests.

## Authors' contributions

LS participated in setting up the design, collected the data, conducted statistical analysis and drafted the manuscript. MS participated in data collection and helped to draft the manuscript. WS set up the design, participated in statistical analysis and helped to draft the manuscript. KM set up the design, participated in statistical analysis and helped to draft the manuscript. All authors read and approved the final manuscript.

## Pre-publication history

The pre-publication history for this paper can be accessed here:

http://www.biomedcentral.com/1471-244X/11/78/prepub
